# Longitudinal changes in the volume of residual lung lobes after lobectomy for lung cancer: a retrospective cohort study

**DOI:** 10.1038/s41598-024-63013-y

**Published:** 2024-05-27

**Authors:** De-Hao Tu, Chong Yi, Qianyun Liu, Lingmei Huang, Guang Yang, Rirong Qu

**Affiliations:** 1 Department of Thoracic Surgery, Yueyang Central Hospital, Yueyang, Hunan China; 2 Department of Pulmonary and Critical Care Medicine, Yueyang Central Hospital, Yueyang, Hunan China; 3 Department of Medical Imaging, Yueyang Central Hospital, Yueyang, Hunan China; 4 Department of Thoracic Surgery, Tongji Hospital, Tongji Medical College, Huazhong University of Science and Technology, 1095 Jie Fang Avenue, Wuhan, 430030 Hubei China

**Keywords:** Lung cancer, Lobectomy, Pulmonary function, Lung volume, Video-assisted thoracic surgery, Lung cancer, Respiration, Surgical oncology

## Abstract

It is unclear how the residual lobe volume changes over time after lobectomy. This study aims to clarify the temporal patterns of volume changes in each remaining lung lobe post-lobectomy. A retrospective review was conducted on patients who underwent lobectomy for lung cancer at Yueyang Central Hospital from January to December 2021. Lung CT images were reconstructed in three dimensions to calculate the volumes of each lung lobe preoperatively and at 1, 6, and 12 months postoperatively. A total of 182 patients were included. Postoperatively, the median total lung volume change rates relative to preoperative values were -20.1%, -9.3%, and -5.9% at 1, 6, and 12 months, respectively. Except for the right middle lobe in patients who underwent right upper lobectomy, the volumes of individual lung lobes exceeded preoperative values. The volume growth of the lung on the side of the resection was significantly more than that of the lung on the opposite side. For left lobectomy patients, the right lower lobe’s volume change rate exceeded that of the right upper and middle lobes. Among right lobectomy patients, the left lower lobe and the relatively inferior lobe of right lung had higher volume change rates than the superior one. Right middle lobe change rate was more in patients with right lower lobectomy than right upper lobectomy. Six months postoperatively, FEV1% and right middle lobectomy were positively correlated with the overall volume change rate. One year postoperatively, only age was negatively correlated with the overall volume change rate. 75 patients had pulmonary function tests. Postoperative FEV1 change linearly correlated with 1-year lung volume change rate, but not with theoretical total lung volume change rate or segmental method calculated FEV1 change. Time-dependent compensatory volume changes occur in remaining lung lobe post-lobectomy, with stronger compensation observed in the relatively inferior lobe compared to the superior one(s). Preoperative lung function and age may affect compensation level.

## Introduction

Although the incidence of lung cancer has dropped to the second among malignant diseases, it remains the leading cause of cancer-related deaths, and its incidence continues to rise^1^. Lung resection is the preferred treatment for early-stage lung cancer. However, due to the inability of the remaining lung to regenerate alveolar units to fully compensate for lost lung tissue, the surgery results in permanent damage to lung function. The damage to lung function can significantly impact the quality of life and even the long-term survival of patients^2,3^. Therefore, accurately predicting the long-term lung function after surgery is crucial for patients undergoing lung resection.

Before performing lung resection surgery, doctors typically measure the patient's lung function indicators, including vital capacity and forced expiratory volume in 1 s (FEV1). Theoretically, the postoperative lung function of patients can be predicted using the number of functional lung segments removed, i.e., predicting postoperative FEV1 = preoperative FEV1 × (1 − the number of functional lung segments intended to be removed / total number of functional lung segments)^4,5^. But many studies have shown that this calculation method underestimates the patient’s long-term lung function^6,7^. These suggest that compensatory mechanisms play a crucial role in the postoperative recovery process for lung resection patients.

Changes in lung volume are closely related to changes in lung function^8–10^. In fact, the increase in lung volume after lung resection is not simply due to overinflation of small airways and alveoli but involves a complex process of vascular proliferation and alveolar structural remodeling^11,12^. In animal models of pneumonectomy, researchers found that the volume and vascular growth of different residual lung lobes are different^12,13^. Considering the unique shape of the human thorax and upright walking, the compensation pattern of the human lung may be different from that of animals. This study aims to investigate the overall trends in volume changes of residual lung lobes over time in patients after various lobectomies.

## Materials and methods

### Ethics approval

This retrospective study was conducted in accordance with the Declaration of Helsinki and approved by the Ethics Committee of Yueyang Central Hospital (approval number: 2023-026). Due to the retrospective nature of the study, the need of informed consent was waived by the Ethics Committee of Yueyang Central Hospital (approval number: 2023-026).

### Patients

We conducted a review of thoracic surgery database at Yueyang Central Hospital, examining all patients who underwent lung resection surgery in our hospital from January to December 2021. Prior to surgery, all patients were subjected to a chest CT scan within a two-week window. Post-surgery, patients who had undergone lung resection due to malignant diseases were advised to have follow-up chest CT scans at the 1, 6, and 12-month marks. Any patients who missed any of these CT scans were excluded from our review. Reasons for exclusion encompassed failure to follow up in a timely manner, undergoing a CT scan at a different hospital, a decision by the doctor to forego a CT scan, or death within a 12-month period. For patients who had undergone pulmonary function test one year after surgery, the results were recorded.

### Data collection and definitions

Clinical and demographic data, such as age, gender, body mass index (BMI), smoking history, comorbidities, history of lung resection, lung function parameters, extent of resection, surgical approach [thoracotomy or video-assisted thoracic surgery (VATS)], pathological type and stage, and postoperative complications, were all collected. Both current and former smokers ceased smoking prior to surgery. Chronic obstructive pulmonary disease (COPD) was defined as an FEV1/forced vital capacity (FVC) ratio of less than 0.7 after the inhalation of a bronchodilator. Overweight was defined as BMI over 24.0. We routinely performed single-port VATS surgeries of 3–4 cm, and when single-port operation proved challenging, we added 1 to 2 auxiliary incisions of about 2 cm each. Cases with more than 3 incisions or a total incision length exceeding 10 cm were classified as open surgeries and were consequently excluded from the study. We routinely performed systematic lymph node dissection, which involved clearing groups 5, 6, 7, 9, 10, and 11 lymph nodes during left lung surgery, and groups 2, 4, 3a, 7, 9, 10, and 11 lymph nodes during right lung surgery. All surgeries were carried out by two senior doctors within the department. Other exclusion criteria included: pulmonary fibrosis, previous lung surgery, intraoperative diaphragmatic nerve injury, and the presence of pneumothorax or pleural effusion requiring drainage or pulmonary infection requiring antibiotic treatment as revealed in follow-up CT scans.

### CT parameters and image processing

Chest CT scanning was implemented using a 16-slice CT system (Lightspeed 16, GE Healthcare) at our institution. The patient was positioned supine. During a deep inspiratory breath hold, we obtained high-resolution CT images that were 1.25-mm-thick and covered the entire lungs in a 512 × 512 matrix. This was achieved using a 20-mm collimation (16 × 1.25 mm), with a rotation time of 0.5 s, at 120 kVp and 100–440 mA. Subsequently, the transaxial CT images were reconstructed using the lung algorithm.

Three-dimensional (3D) lung volume images were created using imaging software Mimics Medical 21 software (Materialise NV, Leuven, Belgium). Firstly, the “Segment Airways” tool was used to semi-automatically segment the airway by indicating the trachea. Then, with the “New Centerline Label” tool, a new centerline was created from the 3D model of the airway and the names of centerline branches was automatically assigned. Next, the “Segment Lung and Lobes” tool was used to segment the lungs and detect the lung separating fissures; subsequently the lungs were cut into lung lobes. The operation creates masks and 3D models of the left and right lung, as well as separate 3D models for each of the lung lobes. The tool relied on both the centerline of airway and a CT threshold to distinguish lung tissue from other thoracic structures. The CT threshold that we used was at the software’s default setting, which was in the range of -1024 to -500 HU. Within this range, localized infections and areas of lung atelectasis were excluded and not included in the lung model; for the gas-containing residual cavity in the thoracic cavity, the software sometimes counted it into the lung volume; we manually removed this part of the model image after the software modeling using the “Cut Thought Points” tool. The division of lung fissures was mainly performed automatically by the software. When the software made a significant error in the recognition of lung fissures, manual corrections were made as the software provided “Add Point” and “Remove Point” tools after detecting lung fissures. When the patient’s lung fissure was underdeveloped, the lung fissure was cut in the middle of the area with the least lung texture without cutting the visible airway on CT. After the procedures, the property of each 3D model could be displayed, which included the volume of the model. All reconstruction operations were completed by the same thoracic surgeon, and all the images were reviewed by a radiologist. The obtained data were jointly recorded by members of the research team.

### Statistical analysis

Statistical analyses were performed using STATA 17.0 software (Stata Corp., College Station, TX, USA). The volume change rate (VCR) is defined as (postoperative lung actual volume/preoperative lung volume) − 1. Theoretical VCR of the total lung is equal to 0 minus the preoperative volume of the resected lobe divided by the preoperative total lung volume. The postoperative FEV1 change rate is defined as (postoperative FEV1/preoperative FEV1) − 1. Theoretical FEV1 change rate is equal to 0 minus the number of functional lung segments intended to be removed/total number of functional lung segments. Continuous variables are presented as median and interquartile range (IQR), and categorical variables are presented as number and percentage. The signed rank test was used for paired continuous variables (e.g., comparing the VCRs of different lobes with the same lobectomy), while the Wilcoxon rank-sum test was used for unpaired continuous variables (e.g., comparing the VCRs of different lobes with the same lobectomy) but different lung lobes for matched continuous variables, and comparing the lung VCRs of patients with different lobectomies). Multiple linear regression analysis was performed to analyse influencing factors of clinical variables. Linear regression analysis was used to test the relationship between the two continuous variables. We first explored potential independent variables to see which of them could have collinearity with each other in the model. All statistical testing was 2-sided, and P values < 0.05 were considered statistically significant.

## Results

### Patients and characteristics

**Figure 1:**
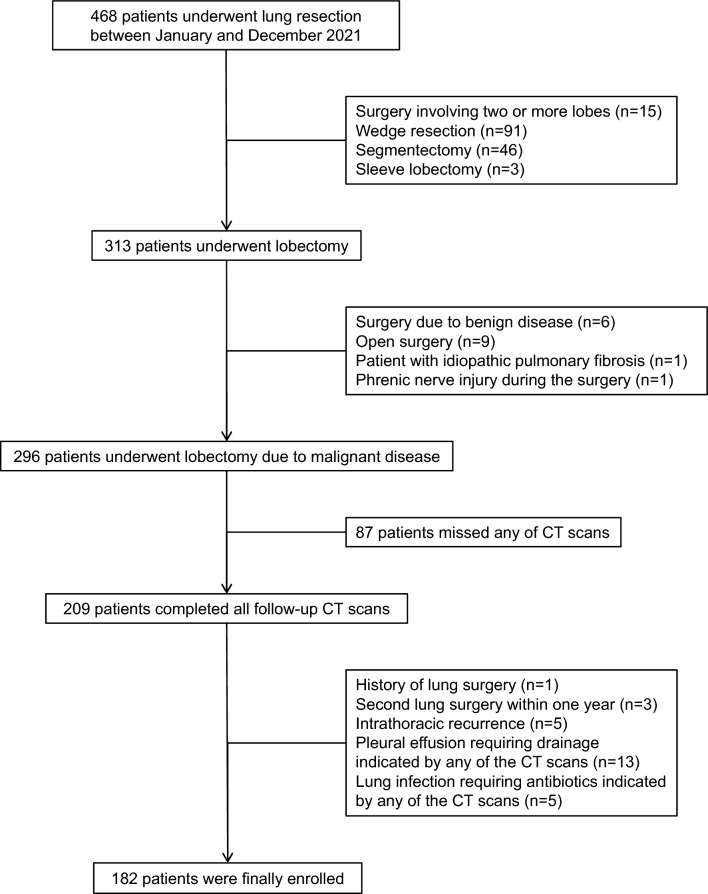
Flowchart of patient selection.

Figure 1 shows the patient selection flowchart. A total of 182 patients who underwent lobectomy due to malignant disease in our institute were finally included in this study. Complete descriptions of participants are listed in Table 1. None of the patients included had undergone neoadjuvant treatment, and none had complications of grade 3 or above according to the Clavien-Dindo system. The volumes of individual lung sections and their respective proportions of the total lung volume are displayed in Table 2.

**Table 1:** Demographics and clinical characteristics.

Variables	Values
Sex	
Male	94 (51.6)
Female	88 (48.4)
Age	58 (51–65)
BMI	22.9 (21.3–24.2)
Overweight	60 (33.0)
Smoker	58 (31.9)
Preoperative lung function	
FEV1 (L)	2.47 (2.02–2.99)
FEV1% (%)	98.3 (91.1–110.2)
FVC (L)	3.18 (2.69–3.84)
FEV1/FVC (%)	78.2 (73.7–81.7)
DLCO (ml/min/mmHg)	6.8 (5.98–8.63)
DLCO% (%)	90.1 (77.0–102.1)
Comorbidities	
COPD	27 (14.8)
Hypertension	43 (23.6)
Cardiovascular disease	25 (13.7)
Diabetes mellitus	15 (8.2)
Pathology	
Adenocarcinoma	159 (87.4)
Squamous carcinoma	18 (9.9)
Others	5 (2.7)
Stage	
I	161 (88.5)
II	9 (4.9)
III	12 (6.6)
Surgery	
Left upper lobectomy	40 (22.0)
Left lower lobectomy	23 (16.6)
Right upper lobectomy	70 (38.5)
Right middle lobectomy	25 (13.7)
Right lower lobectomy	24 (13.2)

Values in the table are presented as median (IQR) or n (%).

*BMI* body mass index, *FEV1* forced expiratory volume in 1 s, *FVC* forced vital capacity, *DLCO* diffusing capacity of the lungs for carbon monoxide, *COPD* chronic obstructive pulmonary disease, *IQR* interquartile range.

**Table 2:** Volume and percentage of each lung section.

Baseline	Volume (mL)	Ratio to total lung volume for each patient (%)
LUL	997.4 (769.7–1268.8)	25.4 (23.6–26.9)
LLL	812.0 (665.3–1053.7)	21.0 (18.5–22.5)
RUL	797.1 (630.2–1083.4)	21.8 (19.2–23.5)
RML	392.1 (284.0–460.4)	9.4 (8.1–10.8)
RLL	955.8 (677.0–1150.7)	23.3 (20.9–25.9)
Left lung	1817.6 (1464.1–2282.7)	45.9 (44.5–47.5)
Right lung	2177.7 (1667.2–2704.4)	54.1 (52.5–55.5)
Total lung	3936.8 (3067.5–4981.4)	1

Values are presented as median (IQR).

*LUL* left upper lobe, *LLL* left lower lobe, *RUL* right upper lobe, *RML* right middle lobe, *RLL* right lower lobe, *IQR* interquartile range.

### Changes in remaining lung volume after lung lobectomy

**Figure 2:**
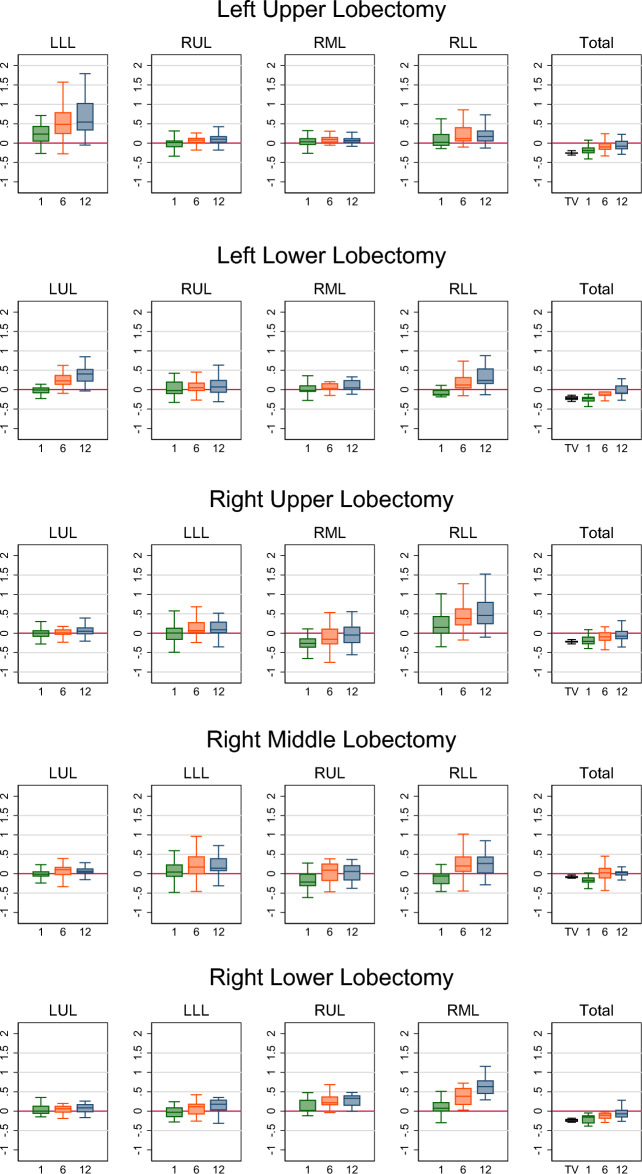
Box plot of lung volume change rates at different postoperative time. The y-axis represents the volume change rate, which is defined as: postoperative actual volume/preoperative volume − 1. The x-axis represents time [month(s)]. TV: theoretical volume, whose change rate is defined as 0 − preoperative volume of the resected lobe/preoperative total lung volume. *LUL* left upper lobe, *LLL* left lower lobe, *RUL* right upper lobe, *RML* right middle lobe, *RLL* right lower lobe, *IQR* interquartile range.

For the entire cohort, the median overall VCRs at 1, 6, and 12 months postoperatively were − 20.1% (− 28.2 to 10.7%), − 9.3% (− 18.4 to 1.8%), and − 5.9% (− 13.7 to 6.0%), respectively. There was no statistical difference between the overall theoretical VCR and the VCR at the first month after surgery (P = 0.4149), but the VCRs at 6 and 12 months postoperatively were significantly greater than the theoretical VCR (both P < 0.001). As shown in Fig. 2 and Table 3, patients' total lung volume exhibited time-dependent compensatory changes within one year after respective lung lobectomy. However, except for patients who underwent right middle lobectomy, the majority of patients who had other lobes removed showed a lower total lung volume after surgery compared to preoperative levels.

**Table 3:** Volume change rate of various parts of the lung at one year after surgery.

Change rate (%)	Left upper lobectomy	Left lower lobectomy	Right upper lobectomy	Right middle lobectomy	Right lower lobectomy
LUL	− 100	40.4 (20.7 to 53.1)	4.8 (− 2.8 to 14.8)	6.4 (1.8 to 13.7)	8.5 (− 2.6 to 18.2)
LLL	54.3 (32.5 to 103.3)	− 100	9.0 (2.9 to 29.7)	14.2 (6.4 to 39.7)	17.7 (2.0 to 29.8)
RUL	10.0 (1.2 to 18.2)	6.8 (− 7.9 to 24.9)	− 100	5.9 (− 17.2 to 21.9)	33.1 (13.9 to 40.8)
RML	6.8 (1.1 to 12.5)	4.7 (− 1.2 to 24.4)	− 4.6 (− 25.3 to 16.8)	− 100	63.4 (44.2 to 79.3)
RLL	17.3 (4.3 to 32.7)	23.7 (14.8.0 to 54.7)	46.0 (23.4 to 80.6)	26.5 (0.6 to 44.2)	− 100
Left Lung	− 28.3 (− 38.7 to 12.6)	− 30.6 (− 33.7 to 22.0)	6.8 (− 0.8 to 22.1)	9.4 (4.1 to 25.0)	11.8 (2.5 to 22.1)
Right Lung	14.7 (3.1 to 21.8)	12.4 (7.1 to 35.2)	− 18.4 (− 27.4 to 5.0)	− 10.2 (− 13.5 to 1.8)	− 23.9 (− 27.4 to 11.8)
Total Lung	− 8.1 (− 16.0 to 5.9)	− 8.9 (− 11.0 to 10.8)	− 7.6 (− 15.3 to 5.8)	2.6 (− 4.3 to 6.0)	− 7.1 (− 16.3 to 3.2)

Values are presented as median (IQR).

*LUL* left upper lobe, *LLL* left lower lobe, *RUL* right upper lobe, *RML* right middle lobe, *RLL* right lower lobe, *IQR* interquartile range.

### Compensatory patterns of remaining lung lobes after lobectomy

In general, most patients consistently experienced volume increase in the retained lung lobes within one year, resulting in final volumes higher than the preoperative levels. However, except for patients who had undergone upper right lobe resection, 52.86% of them had consistently lower volumes in the middle lobe after surgery compared to the preoperative levels (Fig. 2 and Table 3). Among patients who underwent right upper lobectomy, there was no statistically significant difference in the proportion of preoperative RUL volume to total lung volume between those who experienced an increase in middle lung volume and those who experienced a decrease in middle lung volume one year after surgery [22.2% (19.8–23.8%) vs 21.7% (19.9–23.9), P = 0.564]. There was no statistically significant difference in VCR of RLL between those who experienced an increase in middle lung volume and those who experienced a decrease in middle lung volume one year after surgery [44.2% (27.7–66.4%) vs 68.6% (20.1–88.8), P = 0.438].

For patients undergoing left lobectomy, the change rate of the remaining left lung was higher than that of the right lung [47.8% (29.5–82.7%) vs 13.1% (5.6–25.6%), p < 0.001]; the change rate of the right lower lobe (RLL) was higher than that of the right upper lobe (RUL) [21.6% (7.9–45.0%) vs 9.0% (− 2.8 to 19.8%), p < 0.001], and higher than that of the right middle lobe (RML) [21.6% (7.9–45.0%) vs 6.3% (0.3–13.7%), p < 0.001]. The change rates of the RUL and RML did not show a statistically significant difference [9.0% (− 2.8–19.8%) vs 6.3% (0.3–13.7%), p > 0.999].

**Figure 3:**
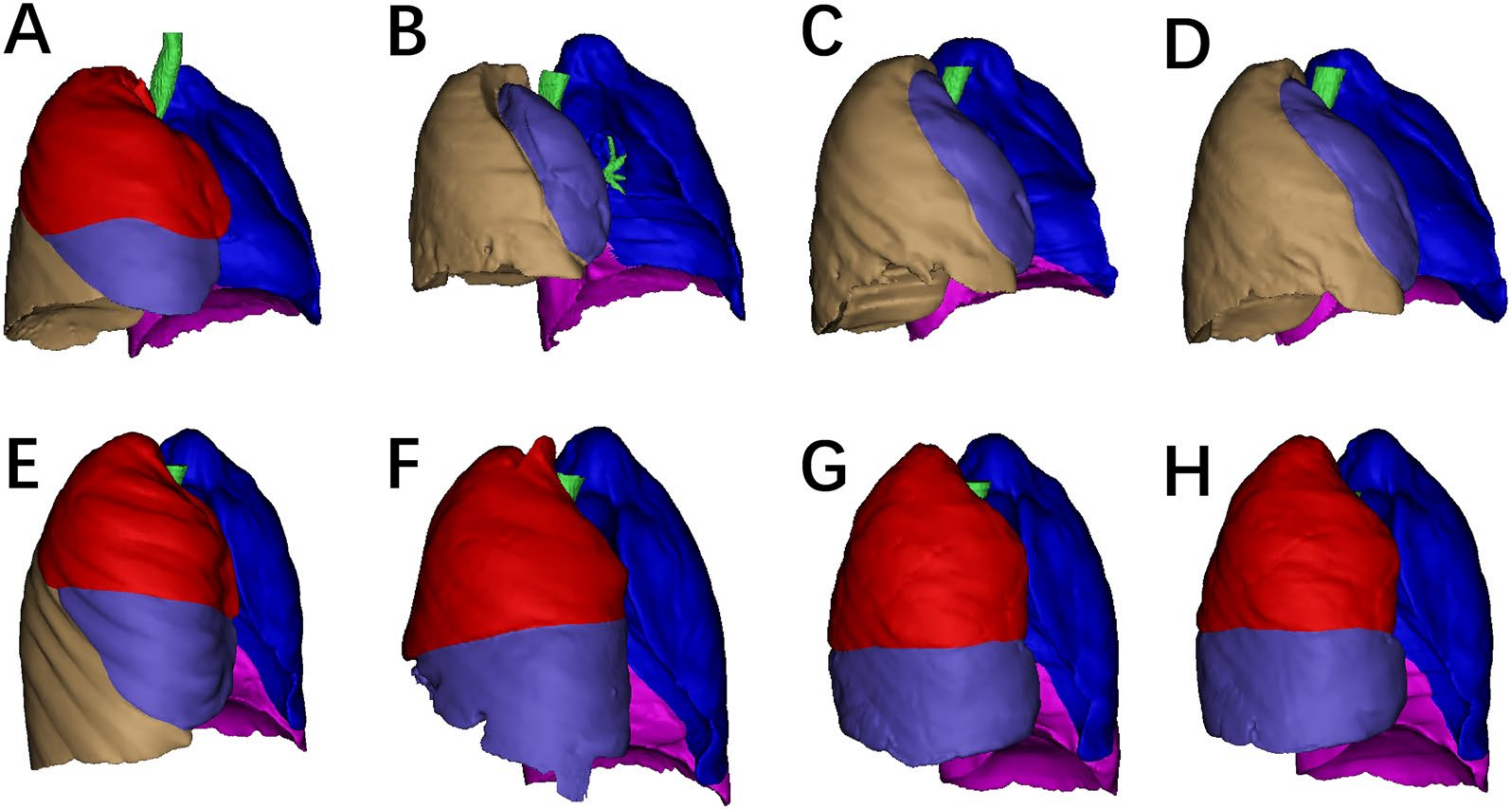
Typical morphological changes before (**A**) and 1 month (**B**), 6 months (**C**), and 12 months (**D**) after right upper lobectomy, and before (**E**) and 1 month (**F**), 6 months (**G**), and 12 months (**H**) after right lower lobectomy.

For patients undergoing right lobectomy, the overall change rate of the remaining right lung was higher than that of the left lung [34.2% (13.2–54.4%) vs 8.7% (− 0.3–22.4%), p < 0.001]; the change rate of the left lower lobe (LLL) was higher than that of the left upper lobe (LUL) [11.7% (0.7–31.5%) vs 6.0% (− 1.8–14.8%), p < 0.001]. There was a greater change rate observed in the relatively inferior lobe compared to the superior one in the right lung [i.e., after right upper lobectomy, the RLL showed more growth than the RML [46.0% (23.4–80.6%) vs − 4.6% (− 25.3–16.8%), p < 0.001]; after right middle lobectomy, the RLL showed more growth than the RUL [9.4% (4.1–25.0%) vs 5.9% (− 17.2–21.9%), p = 0.004]; after right lower lobectomy, the RML showed more growth than the RUL [63.4% (44.2–79.3%) vs 33.1% (13.9–40.8%), p = 0.007]. There was a statistically significant difference in RML VCRs between patients who underwent right upper lobectomy and those who underwent right lower lobectomy [− 4.6 (− 25.3 to 16.8) vs 63.4 (44.2–79.3), p < 0.001] (Fig. 3).

### Factors affecting the VCR of total lung

**Figure 4:**
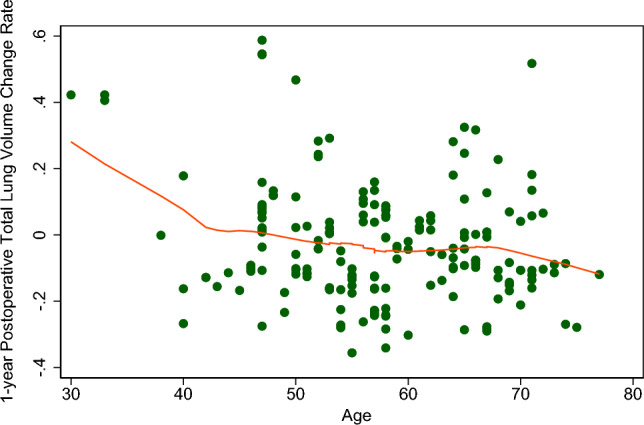
The locally weighted scatterplot smoothing graph of 1-year postoperative total lung volume change rate on age.

Table 4 presents the results of multivariable analysis of the factors associated with the VCR of total lung at different postoperative time. At 1 month postoperatively, none of the factors was found to be related to the overall VCR; at 6 months postoperatively, FEV1% was significantly positively correlated with the overall VCR, and right middle lobectomy was highly correlated with the overall VCR; at 1 year postoperatively, only age was found to be significantly negatively correlated with the overall VCR. The overall VCR one year after surgery for patients under 51 years old, 51–60 years old, 61–70 years old, and over 70 years old were 1.5% (− 11.4 to 11.9%), − 7.2% (− 16.5 to 5.3%), − 6.4% (− 11.8 to 3.2%), and − 10.5% (− 12.8 to 6.2%) respectively. The locally weighted scatterplot smoothing graph of overall VCR one year postoperatively on age was shown in Fig. 4. Patients who are overweight tend to have worse overall VCR at 1 year postoperatively, but the difference is not statistically significant [− 10.2% (− 13.6–4.2%) vs. − 3.8% (− 14.2–6.6%), P = 0.216].

**Table 4:** Multiple linear regression analyses of factors affecting on the volume change rate of total lung at different postoperative time.

Variables	1 month			6 months			12 months		
	Coefficient	Std. err	P-value	Coefficient	Std. err	P-value	Coefficient	Std. err	P-value
Sex (male)	0.0515	0.0291	0.080	0.0570	0.0318	0.074	− 0.0023	0.0348	0.947
Age	− 0.0012	0.0014	0.393	− 0.0017	0.0015	0.263	− 0.0057	0.0016	0.001
BMI	− 1.4169	48.3100	0.977	− 44.4385	52.6057	0.399	− 3.4476	57.6572	0.952
Preoperative FEV1% (%)	0.0005	0.00067	0.469	0.0025	0.0008	0.001	− 0.0002	0.0008	0.852
Smoker	0.0424	0.0305	0.167	0.0298	0.0332	0.371	0.0580	0.0364	0.113
Hypertension	0.0167	0.0274	0.543	0.0404	0.0299	0.178	0.0436	0.0327	0.185
Cardiovascular disease	− 0.0475	0.0341	0.166	− 0.0523	0.0372	0.162	0.0060	0.0407	0.884
Diabetes mellitus	0.0202	0.0437	0.644	− 0.0048	0.0475	0.920	0.0460	0.0521	0.378
Surgery									
Left lower lobectomy	− 0.0182	0.0405	0.653	0.0009	0.0441	0.983	0.0312	0.0483	0.519
Right upper lobectomy	0.0018	0.0296	0.952	− 0.0416	0.0322	0.198	0.0033	0.0353	0.924
Right middle lobectomy	0.0504	0.0399	0.208	0.1100	0.0434	0.012	0.0721	0.0476	0.132
Right lower lobectomy	− 0.0312	0.0400	0.437	− 0.0762	0.0435	0.082	− 0.0470	0.0477	0.326

*BMI* body mass index, *FEV1* forced expiratory volume in 1 s, *Std. err.* standard error.

### Pulmonary function and lung volume

**Figure 5:**
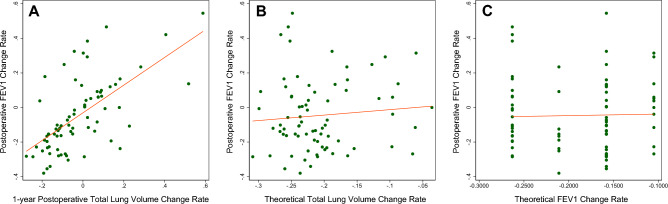
Linear regression analysis of the postoperative FEV1 change rate with the 1-year postoperative total lung volume change rate (**A**), theoretical volume change rate of the total lung (**B**), and theoretical FEV1 change rate calculated by the segmental method (**C**). *FEV1* forced expiratory volume in 1 s.

Among the 182 patients who were studied, 75 had undergone pulmonary function tests one year after surgery. The postoperative FEV1 change rate had a linear relationship with the 1-year postoperative total lung VCR (Y = 0.801X − 0.301; adjusted R-squared = 0.391, P < 0.001; Fig. 5A). The postoperative FEV1 change rate had no linear relationship with the Theoretical VCR of the total lung (Y = 0.316X + 0.019; adjusted R-squared = − 0.005, P = 0.431; Fig. 5B). The postoperative FEV1 change rate had no linear relationship with the Theoretical FEV1 change rate that was calculated by the segmental method (Y = 0.090X − 0.290; adjusted R-squared = − 0.013, P = 0.831; Fig. 5C).

## Discussion

In this study, we investigated the changes in the volume of the remaining lung lobes over time within one year after lobectomy due to malignant tumors. We found that the compensatory growth of the volume of each lobe was significant, although the compensatory capacity of each lobe was not exactly the same.

In China, the Mimics Medical software is widely used for preoperative planning of lung resection. Through three-dimensional reconstruction, this software helps surgeons understand the anatomical variations of the airways and blood vessels inside the lung, and predicts the distance between the tumor and the cutting edge^14–16^. Some previous studies have also used similar three-dimensional reconstruction software to calculate lung volume, and the results showed that there was a significant correlation between the lung volume calculated and the lung function parameters^8–10,17^.

Changes in lung function parameters after lung resection have been widely studied. In the early stages following surgery, the traditional segmental method tends to overestimate a patient’s FEV1 measurement. However, the gap between the actual and predicted values quickly narrows, with the actual value surpassing the predicted one within a span of 3 months^18,19^. Our data suggested that the traditional segmental method could not accurately predict lung function one year after surgery, but the actual change in lung volume after surgery was closely related to the change in lung function. In our cohort, the total lung volume and the theoretical volume of the remaining lung were comparable one month postoperatively. But this included two opposite situations: first, a subset of patients immediately showed significant volume expansion in the ipsilateral lung after surgery, which was more common in the left lower lung of patients with left upper lobectomy and the right lower lung of patients with right upper lobectomy; second, some patients still had a small amount of pleural effusion and localized atelectasis, potentially resulting in the overall lung volume being below the theoretical value. As for the recovery of long-term lung function, Shibazaki et al. conducted a retrospective study on 104 patients who underwent VATS lobectomy and found that the average values of FEV1 at 3, 6 and 12 months after surgery were 85.8%, 87.9% and 89.2% of the preoperative values, respectively^6^. Shin et al. reported in a prospective study that the average values of FEV1 at 2 weeks, 6 months and 12 months after lobectomy 75.7%, 86.7% and 89.8% of the preoperative values, respectively^3^. This is consistent with the patterns of lung volume changes we observed, where rapid recovery occurred within six months and slow recovery between six months and one year after substantial reduction in lung tissue due to the surgery.

Based on existing reports regarding the impact of different types of lobectomy on lung function, except for the RML, there are no significant differences in the long-term effects on lung function parameters following resections of various lung lobes^6,7,9^. For this phenomenon, our study may provide a more detailed explanation. Firstly, according to the principles of the traditional segmental method, the LUL, LLL, RUL, RML, and RLL should contribute 26.3%, 21.1%, 15.8%, 10.5%, and 26.3% of lung function, respectively^4,5^. However, the actual proportion of the RUL is not as small as predicted. Shibazaki et al.^17^ also found that 3D-CT volumetry could predict postoperative FEV1 independent of the resected lobe when predicting postoperative lung function, but the subsegment counting method could not. Secondly, although the volume of the RUL is relatively small, the volume of the RML shrinks after the right upper lobectomy, resulting in a greater actual volume loss than the volume of the RUL itself. Thirdly, compensatory growth of the lungs occurs not only in the ipsilateral lung but also in the contralateral lung. Therefore, even if there is less residual lung tissue on the ipsilateral side, it can be compensated by the growth of the contralateral lung.

The phenomenon of reduced volume in the RML after right upper lung lobectomy may share similarities with some situations in segmentectomy. Nomori et al.^20^ demonstrated that left upper division segmentectomy (which is functionally equivalent to right upper lobectomy) leads to only marginal improvement in lung function parameters compared to lobectomy. Additionally, SPECT imaging revealed that the lingular segment preserved during segmentectomy did not function optimally. Tane et al.^21^ also reported that left S1 + 2 and upper division segmentectomy caused more lung function loss than lingular segmentectomy. Though the volume of segments which had been expected to be rescued by segmentectomy were usually less than theoretical value^10^, Yoshimoto et al.^22^ reported that segmentectomy of RUL caused less function loss of RML compared with right upper lobectomy, which was reportedly caused by less displacement of the RML after segmentectomy than after lobectomy. Our data suggests that the reduction in middle lung volume seems unrelated to the original size of the RUL. Our center typically did not tie the RML and RLL together in right upper lobectomy. Thus, we speculate that the reasons for reduced volume in the RML include: 1. Many patients exhibit incomplete horizontal fissure development, and among these patients, interlobar veins often exist between the RUL and RML. When using a linear stapler to cut the fissure, the staples directly compress, leading to a reduction in the volume of the middle lobe. Simultaneously, interlobar veins may be damaged or severed, hindering blood reflux in the middle lobe and thus affecting its growth. 2. After the RUL is resected, the remaining blood vessels and bronchi of the middle lobe are in a twisted state due to the loss of support. Ueda et al.^23^conducted postoperative CT with airway three-dimensional reconstruction in 50 patients who underwent upper lobe resection, revealing that 42% of patients exhibited bronchial kinking. Disruption of airflow in the airway due to this reason may impact compensatory lung growth^24^. 3. The expansion of the lower lobe, combined with the original shape constraints of the thoracic cavity, transforms the middle lobe into a flat and elongated shape (Fig. 3), limiting its volume increase.

Furthermore, we found that the lung in the relatively lower part of the thoracic cavity always has a stronger volume compensation ability than the lung above. The growth of the residual lung following pulmonary resection is primarily initiated by mechanical stimuli. This includes not only the direct mechanical force exerted by the negative pressure in the thoracic cavity on the alveoli but also the increased blood flow within the remaining vessels due to the reduction in the vascular bed^11,25,26^. In humans, the lungs relatively positioned downward experience not only a more direct and sustained influence from the contraction force of the diaphragm but also a relatively richer blood flow compared to the superior ones.

We used multiple linear regression to analyze the impact of various factors on the overall VCR. As mentioned earlier, in the early postoperative period, various confounding factors might have affected the total lung volume, so no factors that were studied were found to be significantly related to the overall VCR. Six months after surgery, FEV1% and right middle lobectomy were found to have significant correlation with the rate of lung volume change, which might suggest that patients with better preoperative lung function and less resected lung tissue were more likely to have achieved better recovery in the early stage. The overall VCR one year postoperatively was only found to significantly related to age, suggesting that age might be a more important factor affecting the upper limit of lung compensation. This is consistent with previous research^27,28^. Aging itself may lead to reduced lung regenerative capacity, increased alveolar volume, changes in chest wall shape, and decreased respiratory muscle strength^29^, all of which might have long-term effect on lung recovery. Our data could not prove that overweight patients had worse lung volume recovery, despite anecdotal evidence suggesting that overweight patients might experience more pronounced diaphragm elevation. In this study, we only included patients who underwent VATS surgery, partly because over 95% of lung surgeries in our center were performed using thoracoscopy, and open surgery was more closely associated with patients’ specific conditions. On the other hand, Lung surgery with VATS spares more chest wall muscle, cause less surgical trauma and less respiratory muscle injury, than conventional open-chest surgery, and hence are more conducive to postoperative pulmonary function recovery^30^.

This study has several limitations. First, this was a small sample single-center retrospective study, so the clinical characteristics imbalance (such as age, preoperative lung condition, comorbidities, and pathology) among patients undergoing different lobectomies might impact the study results, and the research results were inevitably affected by the basic characteristics of our center’s patients and specific surgical procedures. Second, patients who did not undergo CT scans on time were excluded, many of whom were either relatively non-compliant or changed their follow-up patterns due to special circumstances during the recovery process. Therefore, our analysis may only apply to patients who had relatively good postoperative recovery. Finally, despite our detailed instructions to patients to maintain a deep inspiratory breath hold during CT scans, it was challenging to ensure that all patients consistently maintained the same level of inspiration during each examination, especially when patients experienced postoperative chest pain.

## Conclusions

After a single lobe resection, other lobes can show time-dependent compensatory volume growth changes. Among them, the compensatory growth level of the relatively lower lung is higher than that of the relatively upper lobe. Preoperative lung function and age may be important factors influencing the level of compensation at different times.

## Data Availability

The datasets used during the current study are available from the corresponding author on reasonable request.

## Abbreviations

FEV1: Forced expiratory volume in 1 s
VATS: Video-assisted thoracic surgery
FVC: Forced vital capacity
BMI: Body mass index
COPD: Chronic obstructive pulmonary disease
VCR: Volume change rate
LUL: Left upper lobe
LLL: Left lower lobe
RUL: Right upper lobe
RML: Right middle lobe
RLL: Right lower lobe
